# Women's experiences of early pregnancy assessment unit services: a qualitative investigation

**DOI:** 10.1111/1471-0528.16866

**Published:** 2021-09-07

**Authors:** JA Hall, SA Silverio, G Barrett, M Memtsa, V Goodhart, R Bender‐Atik, J Stephenson, D Jurkovic

**Affiliations:** ^1^ Faculty of Population Health Sciences Elizabeth Garrett Anderson Institute for Women's Health School of Life and Medical Sciences University College London London UK; ^2^ Department of Women & Children's Health Faculty of Life Sciences & Medicine School of Life Course Sciences King's College London St Thomas' Hospital London UK; ^3^ Women's Health Services Elizabeth Garrett Anderson Wing University College London Hospitals NHS Foundation Trust London UK; ^4^ The Miscarriage Association Wakefield UK; ^5^ Gynaecology Diagnostic and Outpatient Treatment Unit Elizabeth Garrett Anderson Wing University College London Hospitals NHS Foundation Trust London UK

**Keywords:** Early pregnancy, pregnancy loss, qualitative research, service evaluation, women's experiences

## Abstract

**Objective:**

To explore the experiences of women who had used an Early Pregnancy Assessment Unit (EPAU) service in the UK and make recommendations for their improvement.

**Design:**

Qualitative interview study.

**Setting:**

Early Pregnancy Assessment Units in the UK.

**Sample:**

A maximum variation sample of women who had consented to be interviewed having attended one of 26 EPAUs involved in the VESPA study in 2018.

**Methods:**

In‐depth telephone interviews with 38 women. A thematic framework analysis was conducted, with a focus on how experiences varied according to EPAU service configuration and clinical pathway.

**Main outcome measures:**

Women's experiences of EPAU services.

**Results:**

We found that EPAUs are highly valued, and women's experiences were generally positive. However, women reported a range of issues that negatively affected their experience. These included difficulties accessing the service, insensitive management of the investigation and treatment options of pregnancy loss, poor communication, insufficient information and a lack of support for their psychological health. These issues were not strongly associated with EPAU configuration or clinical pathway.

**Conclusions:**

Recommendations to improve women's experiences include the separation of EPAUs from general maternity services, and we make suggestions on how to remove barriers to access by reviewing opening hours, how to provide sensitive patient management, such as automatically cancelling appointments and scans following pregnancy loss, and how to improve communication, both with women and their partners as well as with other parts of the health service.

**Tweetable abstract:**

Early Pregnancy Assessment Units are highly valued by women but aspects of their care experiences, particularly around sensitive management of pregnancy loss, could be improved.

## Tweetable abstract

Early Pregnancy Assessment Units are highly valued by women but aspects of their care experiences, particularly around sensitive management of pregnancy loss, could be improved.

## Introduction

Pregnancy can be a turbulent time, both physically and emotionally, for women and their partners. Approximately 30% of women experience bleeding in early pregnancy^1^ and, in the UK, annually, 15% of all clinically confirmed pregnancies end in miscarriage.^2^ Early Pregnancy Assessment Units (EPAUs) have been established across the UK to provide specialist care to women experiencing complications in early pregnancy and, currently, 212 EPAUs operate nationwide.^3^

Most EPAUs have a staff mix consisting of nurse specialists or midwives, trained sonographers and receptionists, whereas some are staffed by doctors. Although consultant gynaecologists are named leads in most EPAUs, they are only timetabled to spend dedicated time in some units. EPAUs differ in terms of the number of women they see each year and their opening hours, but most offer specialist comprehensive assessment of women experiencing early pregnancy complications.^3^, ^4^

Although established in National Health Service hospitals almost three decades ago,^5^ and adopted by other countries,^6^ no formal, national assessment has been undertaken of the variations in service provision and their effect on service‐user views and clinical outcomes.^7^ There was one qualitative study of women’s experience of early pregnancy loss at an EPAU,^8^ but no studies have considered the spectrum of women’s experiences across multiple EPAUs to bring women’s voices to the design of EPAUs, despite recommendations to this effect.^6^

This qualitative study was nested within the larger, multidisciplinary ‘Variations in the organisations of Early pregnancy assessment units in the UK and their effects on clinical, Service and PAtient‐centred outcomes’ (VESPA) study.^9^ This paper explores the service‐user perspective – women’s experiences of attending and being cared for at EPAUs (including exploring whether women’s experiences varied by EPAU configuration or pregnancy outcome) – and uses this information, in addition to women’s own suggestions, to make recommendations to improve service provision and women’s experiences.

## Methods

Women participated in in‐depth interviews discussing their EPAU experience. To achieve a maximum variation sample, the sampling criteria (Table 1) comprised: (1) pregnancy outcome; (2) EPAU configuration (‘strata’: defined on the basis of weekend opening (yes/no), consultant presence (yes/no) and patient volume <2500/≥2500)^9^; (3) geographical location; and (4) Short Assessment of Patient Satisfaction (SAPS) scores^10^ (above or below the median of 25 [out of 28]). Participants had attended one of the EPAUs participating in the VESPA study (26 out of the 44 included) in the preceding 18 months, had completed the VESPA questionnaires containing the SAPS, and had consented to be contacted for interviews. We aimed to conduct around 40 interviews.^11^

**Table 1:** Characteristics of women according to sampling frame

					Total (*n* = 38)
Pregnancy outcome					
Ongoing pregnancy					17
Miscarriage					15
Pregnancy of unknown location					2
Ectopic pregnancy					2
Molar pregnancy					1
Termination of pregnancy					1
EPAU configuration					
*Strata*	*Weekend opening*		*Consultant present*	*Volume of attendances*	
I	No		No	<2500	10
II	Yes		No	<2500	4
III	No		Yes	<2500	5
IV	Yes		Yes	<2500	1
V	No		No	≥2500	4
VI	Yes		No	≥2500	6
VII	No		Yes	≥2500	4
VIII	Yes		Yes	≥2500	4
Geographical location					
East Midlands and The East of England					5
London					6
North West, Yorkshire and The Humber					5
Scotland and The North East					7
South East					7
West Midlands, Wales and South West					8
Short Assessment of Patient Satisfaction (SAPS) Score					
High (≥26)					18
Low (≤25)					20
Clinical care pathways*					
*Pathway*		*Diagnosis, Interventions, and Outcome Descriptors*			
1		Rapid Positive Diagnosis, No Intervention Required, Positive Outcome			15
2		Delayed Positive Diagnosis, No Intervention Required, Positive Outcome			4
3		Rapid Negative Diagnosis, No Intervention Required, Negative Outcome			7
4		Delayed Negative Diagnosis, No Intervention Required, Negative Outcome			5
5		Rapid Negative Diagnosis, Intervention Required, Negative Outcome			5
6		Delayed Negative Diagnosis, Intervention Required, Negative Outcome			3

*Participant 21 appears twice – in ‘Rapid Positive Diagnosis, No Intervention Required, Positive Outcome’ and in ‘Rapid Negative Diagnosis, Intervention Required, Negative Outcome’, because this participant presented to the EPAU twice and received two different diagnoses, the final one of which resulted in loss. Therefore, despite only having 38 participants, a total of 39 clinical care pathways are recorded.

Women were recruited between January and May 2018; contact was made by letter, e‐mail or telephone, with an explanation of the purpose of the study. We contacted women with rarer outcomes (molar/ectopic pregnancies, pregnancies of unknown location and terminations) first, and then completed the sampling frame with women who had undergone miscarriages followed by women with ongoing pregnancies. A total of 153 women were invited to interview, of whom four withdrew, 17 declined, and 93 did not respond before the end of the final response date, leaving 39 women who were interviewed. One interview was excluded because the content did not pertain to an EPAU experience. Telephone interviews were conducted by SAS, a male qualitative research assistant with experience of conducting in‐depth interviews on sensitive topics, who had not met the women. Interviews lasted 20–78 minutes and were digitally recorded with field notes made during and immediately after the interview. Participants were asked about their pregnancy, their experience of the EPAU service, and how they might want services to change or improve in the future (see Topic Guide in File S1). The topic guide and recruitment materials were developed by study team members experienced in qualitative research and reviewed by the wider study team and lay reference group. Participants received a £20 voucher.

All interviews were transcribed and uploaded to QSR nvivo 11 for data management and analysis. The Framework method,^12^, ^13^ which has five steps (familiarisation, developing a thematic framework, indexing [coding], charting, and mapping and interpretation) was used by two authors (SAS and JAH) to analyse data. Initial high‐level codes were created in line with the topic guide and all subsequent codes were created in vivo and discussed between JAH and SAS. Cross‐checking with other members of the VESPA team (VG and GB) enabled maintenance of analytical rigour.^14^ An example of a charting sheet is included in Table S1.

### Patient and public involvement

Recognising that presenting at an EPAU is a time when potential participants are likely to be anxious or distressed, input from former patients and non‐clinical experts was crucial to the planning of patient information and processes. We received input from women who had experienced early pregnancy complications on the design and timing of the study materials through focus groups, patient platforms and surveys, and management oversight through membership of the study steering committee. A user‐led organisation (Miscarriage Association) acted as co‐applicant (through its national director) and collaborator. We carefully assessed the burden and timings of the study participation and questionnaires on patients. There are no plans to disseminate the results of the research directly to the study participants. Dissemination to the population, in general, will be through media outreach (e.g. press release) upon publication and the Miscarriage Association will report it further through their stakeholder networks. Having patient involvement in every part of the VESPA study provided us with the necessary confidence that, despite the sensitive nature, women remained the focus of the study.

### Ethical approval and funding

The VESPA study received ethical approval from the North West Research Ethics Committee (REC reference 16/NW/0587) and was funded by the National Institute for Health Research Health Services & Delivery Research programme grant number 14/04/41. Study registration number ISRCTN 10728897.

## Results

### Sample characteristics and prior survey responses

The sample comprised 38 women. Their median age was 35 years (range 29–45 years) and 32 of them were employed. Most (*n* = 25) identified as white British, but the sample also included women who identified as Asian (*n* = 2), black African (*n* = 2), white other (*n* = 6), mixed (*n* = 1) and other (*n* = 2). The majority were married (*n* = 25) or had a partner (*n* = 12). Women in this study fitted with a range of clinical pathways (see Table 1), which were dependent on their time to diagnosis (‘rapid’ meaning on first visit, ‘delayed’ requiring at least a second visit), their pregnancy outcome (‘positive’ being ongoing, ‘negative’ being any non‐viable pregnancy), and whether they required medical or surgical intervention (in cases of pregnancy loss). Interviewees' SAPS scores, from the original VESPA satisfaction questionnaire, ranged from 11 to 28.

### Overall assessment

In the interviews, women were overwhelmingly positive about the service and the staff. There was widespread recognition of the value of the service; that it was ‘desperately needed’, a ‘valuable asset’ and an ‘important service’ that provided much needed reassurance and management. Seven had nothing negative to say and made no recommendations for change.

With regards to aspects of women’s experience we report seven main themes (Box 1; the full coding tree is in Table S2). We describe each theme and provide quotes in Table 2.

**Table 2:** Quotations

Theme	Illustrative quote(s)	
Barriers	“I'd seen my GP on the Friday and this was the Monday. I had to wait the weekend out basically… But I think I probably would have gone sooner if it hadn't been the weekend.” Participant 013 (Molar Pregnancy, Pathway 6, Strata V)	“The speed of the appointment that I got was very impressive, so that was the most positive thing. Had I had to wait until the following day for an appointment, I think that would have been difficult.” Participant 008 (Miscarriage, Pathway 3, Strata VII)
Efficiency	“We had quite a long wait for the midwife to come through and speak to us about our loss. I mean, it was nearly enough an hour the last time before someone could come and speak to us. So yes, I would say that that did have a slightly negative impact on the quality of care that the staff there could provide. It's out‐with your control, the levels of staffing.” Participant 004 (Ongoing, Pathway 1, Strata VI)	“I think the most positive was once you spoke to them you knew clearly what was happening and the timescale for everything.” Participant 015 (Miscarriage, Pathway 4, Strata IV)
Communication and Information	“I was slightly… confused after that because they simply just said that it wasn't an ectopic pregnancy. The pregnancy was fine, there was no problems with that and that was kind of all the information I was given…I kind of was left a little bit in the dark… it still didn't deal with the symptoms that I'd been having.” Participant 002 (Ongoing, Pathway 1, Strata III) “I remember saying at the time if we could maybe just have a little run‐down of what you expect to happen when you come to an EPAU on the wall or something, at which stage of the process you are actually in, so it would kind of… I felt it would help us to kind of just work through what we were doing and the emotion of it, so we could say, right, we are nearly there.” Participant 023 (ToP, Pathway 1, Strata VI)	“Yes, everything was understood, everything was spoken to us in a language that we understood, not using any jargon, and I think we'll have checked anything at the time just to reassure ourselves. But yes, I came out happy.” Participant 025 (Ongoing, Pathway 1, Strata II)
Involvement in Care Decisions	“…the nurse was explaining to me that, “Look, you can leave it happen naturally or we can keep you in and give you medication,” but it ended up that I didn't really have a choice in the end, they were saying, “Look, we are just going to leave you, leave it to happen naturally”.” Participant 019 (Miscarriage, Pathway 3, Strata VIII)	“I didn't feel like I didn't have a voice, if you know what I mean, so I felt able to discuss things with the staff openly and that was really good. I didn't feel like they were just telling me what to do. I thought they were excellent.” Participant 024 (PUL, Pathway 4, Strata VIII)
Staff Attitudes or Approaches	“From memory, I remember it being quite quick. I don't really know what I was expecting, I've never had a scan before, but I did remember thinking “Oh okay, is that… that's it then, that's that done?” … I do remember thinking “Ooh okay that's quick, we go now?” Participant 034 (Ongoing, Pathway 1, Strata VI) “I wish there was some signs on the wall to say ‘We will be quiet’… When I went to the other unit, they had signs, ‘We'll be quiet when we are looking at the scan’ … it kind of reassures you that actually they are silent for a reason here, but when you are lying in that bed with the silence and you think “what the hell is… please just tell me is everything alright”, that would be beneficial.” Participant 029 (Ongoing, Pathway 2, Strata I)	I think every member of staff that I'd met were very friendly and I just felt very cared for from the moment I went in, so I really would struggle to find a negative as such. But my positive would just be the empathic treatment” Participant 005 (Ongoing, Pathway 1, Strata V) I think they are wonderful, I really do, and I'm glad I'm able to take part in this study to say that because I think without the support and… friendship is not the right word, care, that I've been given, they've made my whole experience from start to finish bearable, really. Participant 012 (Miscarriage, Pathway 3, Strata VIII)
Continuity of Care	“I don't understand why an email can't be sent, at the point that it's clear that you've had a miscarriage, to the GP to let them know that. I don't know why we're still relying on snail mail to get that information to GPs and that's both times that I've had the miscarriage, the information has not made it to the GP by the time that I go to see the GP to get a sick line for my work and that must happen to a lot of people. And certainly the GP said, “I wish that I had been informed,” because he was on the verge of saying congratulations when I went in to see him and had no idea, so again other people might experience that and the doctor does say congratulations to them and it's even more distressing to then have to say, “Well actually I'm here because I've had a miscarriage and I need a sick note”...” Participant 015 (Miscarriage, Pathway 4, Strata IV) “I think the overall system and process was… well, added to anxiety from the point of having to repeat myself so many times about what had happened, sometimes I felt like they didn't really know me or know my situation and that actually if I hadn't have said the right thing I could have ended up with a very different treatment path.” Participant 021 (Miscarriage, Pathway 1 and 5, Strata III) “In terms of mental health, it was hard and I found the three or four months after the miscarriage in particular quite difficult. So, I think if there is support available for that I wasn't aware of any and so I think being made aware of any kind of support there would be helpful.” Participant 008 (Miscarriage, Pathway 3, Strata VII) “It is worth a little bit more sort of signposting at the end. Nobody said you can't see a GP and you can't contact The Miscarriage Association but perhaps just a little bit more explicit reminding you have been through an ordeal and that there are other avenues of support available.” Participant 014 (Miscarriage, Pathway 4, Strata II)	“Even though I was having different people scan me, I think it was the same nurse that was in the quiet room talking to me about the different things. I think I'd seen her two or three times. It was nice to see the same person”. Participant 003 (Miscarriage, Pathway 6, Strata I)
Sensitive Patient Management	“Women going through miscarriages need to be in a slightly more isolated area, they don't need to be walking into a door where there's a woman walking out with her newborn baby, and I think the Early Pregnancy Units, although they are run by midwives, I think they need to be carefully placed, not directly in the line of a labour ward or an antenatal clinic. I think that is so important.” Participant 001 (PUL, Pathway 3, Strata III) “I've been on both sides and it's really sad and it's really hard I suppose to accept when there's people coming out [of having a scan] with big smiles on their faces when they've had a positive picture and yours isn't.” Participant 034 (Ongoing, Pathway 1, Strata VI) “…I had a woman come up to me and try and force me to have the flu jab… you know, when it became recommended for you to have it in pregnancy and I said quite quietly, “I don't need to, I'm here because I'm having a miscarriage,” and she didn't hear, so I ended up having to say it quite loudly and then everyone stared. Yes, it sort of added to what was already a nasty experience.” Participant 006 (Ectopic, Pathway 5, Strata I) “There's a central reception area with a window either side, so the early pregnancy people go to the left and the pregnant people go to the right, so you are in completely different waiting rooms which is a massive plus and I think, where possible, all EPAUs should be set up like that so that you are not sat with other healthy pregnant women.” Participant 012 (Miscarriage, Pathway 3, Strata VIII)	“I think the positive was when they'd realise something was different, they didn't leave me then waiting in the waiting room, a nurse came and they took us into a private room and then we were sitting in there, so we didn't have to stay with all the other people in the waiting room, so that was helpful.” Participant 006 (Ectopic, Pathway 5, Strata I)

Abbreviations: GP, general practitioner; PUL, pregnancy of unknown location; ToP, termination of pregnancy.

Box 1 **Seven main themes describing women's experiences of EPAU services**

**Table:** 

Barriers
Communication & Information
Continuity of Care
Involvement in Care Decisions
Staffs' Attitude or Approach
Efficiency
Sensitive Patient Management

### Barriers

Barriers to attendance included lack of awareness of the service, difficulty obtaining appointments and inaccessibility, in terms of travel and parking at the EPAU. Awareness and accessibility showed no pattern by EPAU configuration, whereas accessibility issues were site‐specific.

Ease of obtaining appointments was the most commonly raised barrier, seen in every EPAU configuration. Availability of appointments (including appointment guarding/blocking by EPAU staff) and opening hours were the main issues raised. Daytime appointments posed a barrier for some, particularly for follow‐up appointments, because of time needed off work, childcare issues and partners' work schedules. Women recommended extended opening hours: almost half of women attending EPAUs that were closed at the weekend reported difficulties obtaining an appointment, compared with less than a third of women attending EPAUs that opened at weekends.

### Efficiency

Although some women reported a swift and smooth journey through the EPAU, others gave examples of inefficient EPAU practices. These included having to repeat the presenting complaint multiple times and long waiting times or delays, either for an appointment or while at the hospital, which were thought to be due to understaffing.

There was no pattern by configuration, but more women in strata II and VI reported ‘good’ efficiency than in other strata. These EPAUs opened at weekends, did not have dedicated consultant presence and only differed by volume of patients they saw (strata II <2500; strata VI >2500).

### Communication and information

Communication and information were deemed sufficient where women reported that they had received information relevant to their condition and/or treatment. This often meant they had not needed to subsequently conduct their own research, and had an opportunity to ask questions, which were answered free of jargon, and where they had not felt rushed through the appointment. Women who were confirmed to have a viable pregnancy at their first visit (clinical pathway 1, see Table 1) were least likely to be satisfied with the communication and information they received. Their dissatisfaction was because they felt that their pregnancy complications and symptoms, or what to expect next, had not been fully explained.

Women enduring the longer clinical pathways with negative pregnancy outcomes involving interventions (clinical pathways 5 and 6), were also more likely to be dissatisfied with the levels of communication and information that they had received, perhaps linked to the greater complexity of their diagnosis and treatment, and therefore showing a greater need for information.

Many women suggested having information in the waiting room covering various things: the process; what the EPAU is; who it is for; information about different conditions; and contact details for relevant organisations.

### Involvement in care decisions

Most women felt that they were actively involved in the decisions regarding their care, and several described how EPAU staff also engaged partners in care decisions.

Only two women reported poor involvement in care decisions. They both had miscarriages but followed different clinical pathways and were in different strata (strata I, clinical pathway 4 and strata VIII, clinical pathway 3, respectively) therefore issues could not be directly linked to either of these. However, in both cases women had been told about available options but had not been allowed to make their own decision.

### Staff attitudes or approach

All women reported good interactions with staff, regardless of clinical pathway or EPAU configuration. Staff were described as kind, compassionate, empathic and competent, often going ‘above and beyond’. Despite this, a third of women did report incidences of negative staff interactions, including describing those performing the scan as being cold, clinical or impolite, or being treated as if they were on a conveyer belt. Some women also reported feeling that their early pregnancy or potential loss was not handled sensitively. One woman with a 7‐week pregnancy said ‘I just felt like I wasn't treated like a pregnancy’ (Participant 019, miscarriage, pathway 3, strata VIII) and another said ‘I might have only been 10 weeks but they were about to cut my baby out.’ (Participant 009, ectopic, pathway 5 strata I). This led to the recommendation of:

… just having a bit of empathy, although they may view it medically as oh it's not a pregnancy, it's only 12 weeks or 8 weeks into a pregnancy, that actually somebody might have pictured a life or imagined having a child with that pregnancy. (Participant 019, Miscarriage, Pathway 3, Strata VIII)

For women this negative experience was most marked at the time of their scan. Explaining, as one woman suggested, that the sonographer requires time to concentrate on the examination could prevent healthcare professionals being perceived as removed or detached while scanning.

Negative experiences were more likely to be reported by women in the longer clinical pathways (4–6) possibly because these women had a greater number of interactions with EPAU staff. Interestingly, participants who reported feeling ‘rushed’ all came from clinical pathway 1, i.e. women with a live pregnancy who did not need any further care at the EPAU, which may reflect their short journey and a mismatch in expectations.

### Continuity of care

There were two types of continuity of care: external (i.e. referral into the EPAU and discharge from the EPAU into other clinical or routine care); and internal (i.e. within the EPAU service). Only six women reported aspects of poor continuity of care. Half of those women attended strata III EPAUs and two of them were at the same one, which suggests there may be a site‐specific issue rather than an EPAU configuration issue. Lack of communication between staff in individual EPAUs, and poor linkages between EPAUs and other hospital departments, GPs and community midwives were issues that transcended strata.

In relation to clinical pathway, the main finding was of the potential additional distress caused if the GP and/or community midwife was not informed of a pregnancy loss. This overlapped with the theme of sensitive patient management. Several women commented on how their ongoing psychological health was inadequately addressed.

### Sensitive patient management

EPAU co‐location with maternity services, privacy and practical sensitivities, such as having to explain why you needed to cancel all your scans, were raised as problematic issues. Women who attended smaller EPAUs (strata I, II, III) were more likely to have poor or mixed experiences of care. These were usually ascribed to privacy issues, such as being aware that everyone in the waiting room could hear what was being said in the consultation room or at reception.

All women, regardless of pregnancy outcome or satisfaction, particularly disliked having EPAUs co‐located with other maternity services. Women experiencing early pregnancy complications did not want to see other pregnant women coming for scans or in labour, or to be offered services for pregnancy that they had to decline. Women made suggestions for how to address this, such as having separate entrances, waiting rooms or pathways through the service.

## Discussion

### Main findings

Our study provides further evidence that EPAUs are a much needed and valued part of the healthcare system, as confirmed by the women who use them. However, like most services, there are ways in which they could be improved. We have provided a platform for women’s voices to articulate and inform these recommendations. We identified seven areas where services could be improved. Issues were rarely found to be strata‐specific or clinical‐pathway‐specific in our data and there were suggestions or examples of best practice from women that all EPAUs could learn from, as summarised in Table 3. Crucially, we also showed that all EPAUs must attend to the sensitivities around actual or threatened pregnancy loss.

**Table 3:** Recommendations

Recommendation	Mapped Themes	“Most Important”	“Quick Wins”
General Pregnancy & Pre‐Pregnancy Care & Information	Barriers	Raise awareness of EPAUs among women of reproductive age, perhaps through a national social media campaign and inclusion in the school curriculum	Provide women with the information they need when they leave the EPAU dependent on their outcome (e.g. amount and duration of bleeding and pain for natural miscarriage and medical miscarriage at home; how long to wait before trying to conceive again, and preconception advice for subsequent healthy pregnancies)
Accessibility of Early Pregnancy Assessment Units	Barriers	Provide suitable opening times, including weekends and evening and ensure alternative care providers for when EPAU is closed	Ensure appointments are not blocked on referral, apart from required checks for appropriateness of attendance Ensure accessible parking for women with early pregnancy complications
Staffing	Efficiency	Ensure EPAUs are appropriately staffed, both in number and skill mix Allocate appropriate appointment times and explain length of and reasons for any delays	Offer women a smooth process through EPAU by passing notes between EPAU staff to prevent re‐explaining symptoms
Within EPAU Experience	Staff Attitudes or Approach	Keep women informed, for example with information on the walls describing the process in the EPAU and that the person doing the scan may not speak while they are working. Be aware that every pregnancy, regardless of gestation, is important to that woman and do not attempt to minimise the loss	
Managing Patients Sensitively	Sensitive Patient Management	Provide a distinct, but integrated EPAU service, by physically separating EPAUs from other hospital services, where possible, but having good cross healthcare links for ongoing care	Emphasise to staff the sensitivity of the nature of EPAU visits and ensure they act accordingly. For example, confirm it is the patient on phone before announcing they are EPAU, reception staff to cancel appointments after a loss without question and prevent routine recruitment, i.e. for flu vaccination taking place in EPAU waiting area Ensure privacy, for example by allowing women to fill out forms rather than verbally state their symptoms and ensuring that consultation rooms are not overheard by providing background noise and a private location
Communicating and Decision Making	Communication & Information Involvement in Care Decisions	Prepare women for the journey ahead of them by providing the information they need to understand and make decisions about what happens when discharged from the EPAU; be that when they go home – whether pregnancy is ongoing or not, or if they are heading to theatre for surgery, onto a ward or to a specialist for medical attention	Provide clear and accessible information that is specific to the condition a woman has and/or procedures they will have Involve women in their care decisions by providing women with the choice of the full range of options, appropriate to their condition and respect their choices if still clinically safe Involve a woman's partner in the processes and procedure, should women want them to be
Continuity of Care	Continuity of Care Sensitive Patient Management	Provide appropriate aftercare by developing new or improving links with psychological support services, in the NHS and/or other support organisations, for women after using EPAU services, giving women information on how to access these services and providing sufficient capacity for timely care. Explain to women the availability of follow‐up with GP, Community Midwife, EPAU or other specialists as appropriate Review referral and discharge processes to ensure a smooth transition into and out of EPAUs to make sure whoever is taking over care after discharge from EPAU is fully informed of women's notes from EPAU	Ensure there is efficient communication between EPAU and rest of maternity and/or hospital care, and community care by sending notes promptly and electronically from EPAU to healthcare providers who will next provide care for women

### Strengths and limitations

This is the first qualitative study to formally investigate experiences of women who have used EPAU services, regardless of pregnancy outcome, in relation to the structure of the EPAU. It contributes to addressing the current paucity of studies of EPAUs adopting a women‐centred approach. We have drawn on a diverse, national sample, with varied pregnancy outcomes, who attended a variety of EPAUs, and who had a range of levels of satisfaction with the care they received. However, because molar and ectopic pregnancies are less common, the number of women with these diagnoses was small in this study (*n* = 1 and *n* = 2, respectively) despite us over‐sampling non‐viable pregnancies. Likewise, although women from all EPAU strata and clinical pathways were represented in the sample, the numbers of interviews per strata/pathway were relatively small. Hence, our findings relating to strata/pathway must be understood as comparisons within our sample, not as definitive reports per strata/pathway. We also did not include the views of partners, who remain under‐researched in this area.

### Interpretation

The few evaluations of EPAUs that do exist have focused on one EPAU,^15^ one aspect of how EPAUs operate, such as multidisciplinary working,^16^ or staffing structures^17^, ^18^ or only on women with pregnancy loss.^8^ No previous studies have brought together all these factors to evaluate women’s experiences with the services they use, making our research unique.

Nevertheless, many of our findings and recommendations are in keeping with the literature around miscarriage. Women have previously described a lack of sympathy, empathy and sensitivity in the management of miscarriage,^19^, ^20^, ^21^, ^22^ particularly during the scan, as we found.^8^ Like the women in our study, they objected to being co‐located with women with ongoing pregnancies,^21^, ^22^, ^23^ had a need for privacy^8^ and described feeling that the loss of their child had not been validated.^21^, ^22^, ^24^, ^25^ Receiving insufficient information, particularly about what to expect in terms of bleeding, pain and duration was a common complaint,^8^, ^20^, ^21^, ^22^, ^25^ and some women noted the lack of choice with regards to their management;^8^ both findings replicated in our study. A lack of formal support following pregnancy loss was also frequently noted and therefore requested,^8^, ^20^, ^21^ a recommendation that is supported by the evidence of the burden of poor mental health in women experiencing miscarriage.^26^, ^27^, ^28^ The uncertainty that women experience when having to wait for an EPAU appointment, for example when they are closed over the weekend, was also raised by another study,^8^ supporting the need to find ways to reduce this barrier to access.

This study was conducted before the coronavirus disease 2019 (COVID‐19) pandemic. The COVID‐19 pandemic has had far reaching indirect impacts through the closure or reduction of non‐COVID‐19‐related healthcare services, including EPAUs. The lack of face‐to‐face care, limited availability of appointments for surgical management and restricted access for partners are likely to have negatively impacted women’s experiences. We hope that EPAU staff and managers are able to view the improvements recommended here as positive and helpful as services return to pre‐COVID‐19 functioning.

## Conclusion

Overall, women were very satisfied with, and valued greatly, the EPAU service. However, some women also described aspects of their experience that could be improved. From our exploration of women’s experience, and using women’s own recommendations, we have developed a set of recommendations for EPAUs to consider, as summarised in Table 3. These include removing barriers to access, both through raising women’s awareness of the service and increasing service accessibility, the latter being in keeping with the NICE guideline that women should be seen within 24 hours of referral to an EPAU.^7^ In addition, by increasing service efficiency, sensitive patient management, communication (both between staff and with patients), continuity of care and (realistic, genuine) involvement in care decisions, women’s experiences could be improved, both by small ‘quick win’ changes and by longer‐term service development, building on the already firm footing of existing services. The separation of EPAUs from other maternity services was a strong and consistently repeated recommendation from women. There are a number of resources available that EPAUs and their staff can use to support the implementation of these recommendations.^29^, ^30^ These recommendations, which vary in their resource‐cost and their ease of implementation, would go to further optimise an essential service, and may be of use to other health services considering how best to care for women with early pregnancy complications. Future research should evaluate the impact of these changes.

### Disclosure of interests

None declared. Completed disclosure of interests form available to view online as supporting information.

### Contribution to authorship

Conceptualisation was by DJ, MM, JS, GB and RBA; methodology was by GB, JAH and SAS; formal analysis and investigation were by JAH and SAS. The original draft was prepared and written by JAH and SAS and reviewing and editing was by all authors. Funding acquisition was by DJ, MM, JS, GB and RBA, and JAH and GB supervised the study.

### Details of ethics approval

The study received a favourable ethical opinion from the North West Research Ethics Committee on 16 September 2016 (REC reference 16/NW/0587).

### Funding

This project was funded by the National Institute for Health Research (NIHR) Health Services & Delivery Research programme grant number 14/04/41. Study registration number ISRCTN 10728897. Sergio A. Silverio (King’s College London) is currently supported by the National Institute for Health Research Applied Research Collaboration South London (NIHR ARC South London) at King’s College Hospital NHS Foundation Trust. The views expressed are those of the authors and not necessarily those of the NIHR or the Department of Health and Social Care.

### Acknowledgements

The authors would like to thank the women who willingly gave their time to participate in the study and shared with us their experiences.

## Supporting information


**Table S1**. An example of a charting sheet.

**Table S2**. Coding tree.

**File S1**. Topic Guide.

Supplementary Material

Supplementary Material

Supplementary Material

Supplementary Material

Supplementary Material

Supplementary Material

Supplementary Material

## Data Availability

Data available on request because of privacy/ethical restrictions.
