# Supplementary material for: Women's experiences of early pregnancy assessment unit services: a qualitative investigation
Source: BJOG. 2021 Sep 7;128(13):2116–25. doi: 10.1111/1471-0528.16866 (PMC9292489; doi:10.1111/1471-0528.16866)
Supplement: Supplementary file 2 — Table S2. Coding tree. [file BJO-128-2116-s005.docx]

VESPA codes

| Name | Files | References |
| --- | --- | --- |
| EXPERIENCES OF EPAU SERVICES | 0 | 0 |
| Barriers | 0 | 0 |
| Awareness of Service | 8 | 8 |
| None | 17 | 19 |
| Obtaining Appointments | 0 | 0 |
| Easy to Obtain Appointments (Initial & Follow-up) | 33 | 71 |
| Problematic Opening Times or Availability of Appointment | 18 | 33 |
| Appointment Guarding or Blocking | 4 | 11 |
| Travel and Accessibility | 0 | 0 |
| Distance | 6 | 6 |
| Parking availability and fees | 13 | 13 |
| Communication & Information | 0 | 0 |
| Insufficient Communication & Information | 13 | 29 |
| Jargon Used | 3 | 4 |
| Patient Conducting Own Research | 8 | 19 |
| Sufficient Communication & Information (Verbal) | 21 | 31 |
| Opportunity to Ask Questions | 14 | 26 |
| Time Taken to Explain (No Jargon) | 31 | 59 |
| Written information | 24 | 40 |
| Experiences of Care | 0 | 0 |
| Continuity of Care | 0 | 0 |
| Consistently Staffed Team | 10 | 19 |
| Integration & Information Sharing | 0 | 0 |
| with GP or ongoing maternity care | 22 | 36 |
| with hospital or AE | 10 | 19 |
| with other EPAUs | 2 | 2 |
| within EPAU | 7 | 14 |
| Involvement in Care Decisions | 0 | 0 |
| Adequately Involved | 15 | 22 |
| Insufficient Involvement | 2 | 5 |
| Involving Partner | 5 | 6 |
| No Decisions to be made | 16 | 23 |
| Patient-led Care | 14 | 24 |
| Staffs' Attitude or approach | 0 | 0 |
| Cold, Clinical, unkind, impolite | 12 | 40 |
| Compassion, Empathy, Respect, & Sensitivity | 38 | 137 |
| Going Above and Beyond | 17 | 35 |
| Not Rushing Patients | 24 | 36 |
| Professional Conduct or Competency | 32 | 76 |
| The EPAU's Functionality | 0 | 0 |
| Efficiency | 0 | 0 |
| Efficiency | 10 | 12 |
| Delays or waiting time | 10 | 13 |
| Understaffed | 8 | 11 |
| Timely Service | 20 | 36 |
| Sensitive Patient Management | 0 | 0 |
| Familiarity, Knowledge, and-or Trust of an EPAU | 3 | 4 |
| Privacy | 11 | 14 |
| Sensitivity (Practical) | 16 | 20 |
| Separate EPAU Service | 15 | 32 |
| RECOMMENDATIONS FOR EPAU SERVICES | 38 | 215 |
| Accessibility | 25 | 78 |
| Access to EPAU | 15 | 21 |
| Opening Times | 13 | 26 |
| Reception, waiting room, Greeting Service or Advice Line | 11 | 26 |
| The Estate | 4 | 5 |
| Broader recommendations | 7 | 10 |
| Changes to Prevent Poor EPAU Experience | 31 | 83 |
| Better Information | 25 | 54 |
| Integration of care | 10 | 20 |
| Provision of Ongoing Support | 0 | 0 |
| Aftercare and Check-ups | 19 | 38 |
| Psychological Support Services | 8 | 26 |
| Specialist referrals | 1 | 1 |
| Separate EPAU Specific Service | 22 | 76 |
| Staffing and Efficiency | 1 | 1 |
| Staff Attitude and Practices | 11 | 17 |
| Staffing to prevent delays | 6 | 10 |
| Importance or awareness of service | 19 | 24 |
| None | 7 | 8 |
