# Supplementary material for: Women's experiences of early pregnancy assessment unit services: a qualitative investigation
Source: BJOG. 2021 Sep 7;128(13):2116–25. doi: 10.1111/1471-0528.16866 (PMC9292489; doi:10.1111/1471-0528.16866)
Supplement: Supplementary file 3 — File S1. Topic Guide. [file BJO-128-2116-s006.docx]

**VESPA Study**

**Women’s Interviews - Topic Guide**

*Thank interviewee for agreeing to the interview*

*Remind interviewee what interview is about:*

Example wording:

You attended the Early Pregnancy Assessment Unit at [hospital name] and you completed a number of questionnaires, one while you were in the unit and some afterwards. You are one of a number of women who said that they’d be happy to talk to us some more – so thank you! What I’d like to do in this interview is to ask you about your experiences of the Early Pregnancy Assessment Unit in a bit more detail, and for you to tell me, in your own words, what happened during your time in the EPAU and about your experiences since then. There are no right or wrong answers – we are interested in the full range of women’s experiences and their opinions.

*Reassure confidentiality*

As we begin, and before we talk in detail about your experiences of the EPAU, may I check that the study information I have is correct – about the EPAU and about you?

**EPAU**

*Check current study information/find out if any changes since completion of questionnaires*

*PROBE*

- - *EPAU unit attended*
  - *EPAU attendance dates/time period?*
  - *Outcome of pregnancy – miscarriage, ectopic, ongoing pregnancy etc*
  - *Management of pregnancy outcome*
  - *Current pregnancy situation (e.g. not pregnant, ongoing pregnancy, pregnant again following pregnancy loss)*

**General background information**

Also, I have some background information about you from the study. May I check that that is still correct?

*Check information/Find out if any changes since completion of questionnaires:*

*PROBE:*

- - *Age*
  - *Marital/partnership status*
  - *Number of children*
  - *Employment*

**Beginning of pregnancy**

Now I would like to ask you a bit more about your pregnancy *[the pregnancy associated with EPAU attendance].* So that I understand the background to your experiences with the EPAU, may I take you back to the very beginning of your pregnancy?

Can you briefly tell me a little bit about around the time you got pregnant?

*[Let interviewee volunteer as much information as possible before probing.]*

*PROBE*

- *Were you wanting to get pregnant then?*

How was your pregnancy confirmed?

*PROBE*

- *Pregnancy testing.*
- *Pregnancy signs/symptoms.*
- *Who had the interviewee told about the pregnancy?*

**EPAU experience**

I would like to ask you about your experiences at the EPAU.

To begin with, what led to your attendance at the EPAU?

*PROBE*

- - *Signs/symptoms that led to EPAU attendance*
  - *How referral/attendance happened?*
  - *Any barriers/hurdles in attending (e.g. practical, financial)*
  - *Any issues in decision-making? Health professionals involved?*

Once you were at the EPAU, what happened?

*PROBE (as appropriate)*

- - *The phases of the patient journey*
  - *Staff spoken to*
  - *Verbal information received*
  - *Written information received*
  - *Procedures (e.g. urine pregnancy test, ultrasound, blood tests)*
  - *Options for care offered*
  - *Any decisions made by interviewee (e.g. management method, disposal)*
  - *The factors shaping their decisions (e.g. advice, time, practical considerations)*
  - *Interviewee’s feelings and concerns at each phase*

**EPAU experience - *continued***

Thinking overall about your time in the EPAU, what do you feel about the quality of care that you received?

*PROBE (as appropriate)*

- *Perception of clinical competence of care*
- *Empathy of staff*
- *Involvement in decisions*
- *Information received – appropriateness/relevance/comprehensibility*

Thinking overall about your time in the EPAU, what would you say were the….

- The most positive aspects of the care you received
- The most negative aspects of the care you received

**After the EPAU**

What happened next, after the EPAU?

*PROBE (as appropriate)*

- - *Admissions/referrals*
  - *Experience of management method/procedures*
  - *The match between experience of management method etc and the information received in the EPAU*
  - *Perceptions of quality of care*
  - *Interviewee’s feelings and concerns throughout*
  - *Follow up*

**EPAU services in future**

As you know, this study is about finding out the best way to run EPAU services. Given your experiences, are there any changes to EPAU services that you would recommend?

*PROBE (as appropriate)*

- *Changes to EPAU service organization?*
- *Changes to information provision?*
- *Changes to follow up?*
- *Other changes?*
- *Most important change interviewee would like to see?*

*Thank interviewee*
